# Supplementary material for: Factors associated with variation in single-dose albendazole pharmacokinetics: A systematic review and modelling analysis
Source: PLoS Negl Trop Dis. 2022 Oct 28;16(10):e0010497. doi: 10.1371/journal.pntd.0010497 (PMC9662735; doi:10.1371/journal.pntd.0010497)
Supplement: S1 File — (DOCX) [file pntd.0010497.s001.docx]

**S1 File: Additional Methods and Results**

**Factors associated with variation in single-dose albendazole pharmacokinetics: A systematic review and modelling analysis**

**Short title: Review and modelling of albendazole pharmacokinetics**

Charles Whittaker^1,*^**,** Cédric B. Chesnais^2^, Sébastien D.S. Pion^2^, Joseph Kamgno^3^, Martin Walker^4,5^, Maria-Gloria Basáñez^1,5, §^ & Michel Boussinesq^2, §^

^1^MRC Centre for Global Infectious Disease Analysis, Department of Infectious Disease Epidemiology, School of Public Health, Imperial College London, London, UK

^2^Recherches Translationnelles sur le VIH et les Maladies Infectieuses (TransVIHMI), University of Montpellier, Institut de Recherche pour le Développement (IRD), Institut National de la Santé et de la Recherche Médicale (INSERM), Montpellier, France

^3^Centre for Research on Filariasis & other Tropical Diseases, and Faculty of Medicine and Biomedical Sciences, University of Yaoundé I, Yaoundé, Cameroon

^4^Department of Pathobiology and Population Sciences, Royal Veterinary College, Hatfield, UK

^5^London Centre for Neglected Tropical Disease Research, Department of Infectious Disease Epidemiology, School of Public Health, Imperial College London, London, UK

^§^ Joint senior authors

* [charles.whittaker16@imperial.ac.uk](mailto:charles.whittaker16@imperial.ac.uk)

**Contents:**

**Text A: Data Extraction, Collation and Initial Processing**

**Table A: Studies collated through the systematic review and their associated metadata**

**Text B: Model Construction, Fitting and Inference**

**Figure A: Results of model fitting and calibration to data collated through the systematic review**

**Table B: Multivariate linear regression results relating pharmacokinetic properties to study characteristics when controlling for dosage per kilogram of body weight instead of raw dosage amount in milligrams.**

**Supplementary References**

**Outline of Document**

In this **Supplementary S1 File** we describe the methods and data used to explore and analyse the drivers of variation in albendazole sulfoxide pharmacokinetics. In **Text A: Data Extraction, Collation and Initial Processing**, we present further information on the systematic review conducted, including details of the collated references and information on the metadata (population characteristics, infection status, co-administration of other drugs, etc.) available for each study. In **Text B: Model Construction, Fitting and Inference**, we detail the mathematical and statistical methodologies employed to process these extracted data, whose output forms the basis for the results presented in the Main Text. This includes further details on the pharmacokinetic model, the Bayesian fitting procedures and the multivariate linear regression analysis relating results from the fitting to study metadata. **Table A**, **Figure** **A** and **Table B** present additional results to support the work detailed in the Main Text. Finally, the **Supplementary References** provide details on the papers listed in **Table A** in addition to other references mentioned in this document.

**Supplementary Text A: Data Extraction, Collation and Initial Processing**

***Systematic Review References & Associated Metadata***

We searched the Web of Science and PubMed databases on 22nd July 2022 with no date constraints using the keywords “albendazole” AND (treatment* OR dose* OR pharma* OR “half-life” OR “half life”) in order to identify references containing temporally disaggregated data detailing plasma concentration of albendazole and/or albendazole sulfoxide following treatment with a single dose of the drug. References were selected for Inclusion/Exclusion according to the following criteria:

**Inclusion Criteria:**

- Reference contains data from human subjects describing the plasma concentration of albendazole and/or albendazole sulfoxide following receipt of a single, orally administered dose of albendazole.

**Exclusion Criteria:**

- The study was carried out in animals or *in vitro*, i.e. not in humans.
- The study administered multiple doses of albendazole and does not contain information on plasma drug concentration following receipt of the very first dose.
- Reference does not contain temporally disaggregated information on plasma albendazole/albendazole sulfoxide concentrations.
- The article is not in English.

A total of 9,027 records were identified, with 2,172 duplicates being excluded, leaving 6,855 unique records being retained for title and abstract screening. Title and abstract screening excluded 6,609 references, leaving a total of 246 articles for full-text screening. Studies lacking the required information on plasma concentration levels over time, not in English, that utilised non-standard formulations of albendazole or that had been carried out *in vitro* or in non-human subjects were subsequently excluded. A total of 36 references were subsequently retained and included for data extraction. For each reference, we extracted all relevant albendazole and albendazole sulfoxide concentration data over time that were available, yielding 113 time-series describing the evolution of plasma concentrations of albendazole (n=19) and/or albendazole sulfoxide (n=94) in individuals or groups of individuals following treatment with a single dose. For each time-series, we also extracted relevant metadata and characteristics of the individual/group of individuals receiving treatment. These metadata were:

- **Sex:** The sex of the individual, or composition of sexes in the case of groups of individuals. This was subsequently converted into a categorical variable based on the collated responses, with levels “Males” (where the entire population sample consisted of male subjects), “Mixture” (where the population sample was a mixture of males and females) for use in the regression analyses, and “Females”.
- **Age:** The age of the individual, or in the case of groups of individuals, the mean age of the individuals. This was subsequently converted into a binary indicator according to whether the age of individuals was ≥18 (“Adults”) or <18 years (“Children”), for use in the regression analyses.
- **Dose Amount:** Both the total dose amount (in mg) and the dose per kilogram of bodyweight. Where the latter was not directly provided but the weight of participants was provided, the dose per kilogram of bodyweight was calculated manually.
- **Feeding State:** Whether or not the individual or group of individuals had received a fatty/oily meal prior to receiving albendazole.
- **Co-Administered Drugs:** Details on whether or not the reference reported any drugs that either 1) were co-administered alongside albendazole or 2) which the individuals were receiving prior to receiving albendazole, and continued to take following receipt of the albendazole dose. This was subsequently converted into a binary indicator denoting whether or not any drugs were being taken alongside albendazole (“Yes”/“No”).
- **Infection Status:** Details on whether or not the reference reported that the individual or group of individuals receiving albendazole were doing so because they currently had a parasitic infection (as defined by the reference) and, if so, what parasite species they were infected with. As with co-administered drugs, this was also converted into a binary indicator denoting whether or not the individual or group of individuals had a reported parasitic infection. When information on parasite species was given, the following infections were analysed: neurocysticercosis; echinococcosis; soil-transmitted helminthiases; onchocerciasis; lymphatic filariasis, and giardiasis.
- **Weight:** Where available, we also collated and extracted information on the weight of an individual, or the average weight of a group of individuals.

We extracted albendazole and albendazole sulfoxide plasma concentration data at the individual level where possible, only extracting this information for groups of individuals where individual-disaggregated data were not available. Where data were presented at the individual level but only group-level characteristics (such as age, sex or weight) were presented, we associated each individual-level time-series with the relevant group-level average characteristic. Where individuals had received multiple doses of albendazole but where there were pharmacokinetic data describing plasma concentrations following the first dose, we extracted information on plasma concentrations for all time-points up until receipt of the second dose.

**Supplementary Table A: Studies collated through the systematic review and their associated metadata.** Further details and disaggregation of metadata by each specific time-series (rather than reference) are also available here: <https://github.com/cwhittaker1000/albendazole_pk>.

| **First Author [Ref]** | **Year** | **# Time-Series** | **Number of People** | **Plasma Concentration Information** | **Dose (mg)** | **Dose (per kg)** | **Formulation** | **Sex** | **Age**  **(years)** | **Fatty Meal?** | **Co-Drugs** | **Infection** |
| --- | --- | --- | --- | --- | --- | --- | --- | --- | --- | --- | --- | --- |
| Awadzi  [1] | 2003 | 2 | 28 | AlbSO Only | 400 | Various | Oral Tablet (No Further Info) | All Males | Various (Adults) | None | Various (None; IVM, DEC & PZQ) | Onchocerciasis |
| Awadzi  [2] | 2004 | 1 | 22 | AlbSO Only | 400 | 7.49 | Oral Tablet (No Further Info) | All Males | 43 (Adults) | None | Levamisole | Onchocerciasis |
| Awadzi  [3] | 1994 | 2 | 28 | AlbSO Only | 1200 | NA | Oral (No Further Info) | All Males | NA (Adults) | Various | None | Onchocerciasis |
| Ceballos  [4] | 2018 | 1 | 8 | Alb & AlbSO | 400 | 6.25 | Oral (No Further Info) | Mixture | NA (Adults) | None | None | None |
| Ceballos  [5] | 2021 | 6 | 36 | AlbSO Only | 400 | NA | Oral Tablet (Brand=Zentel) | Various (All Male & All Female Groups) | NA | Various | None | None |
| Chen  [6] | 2004 | 1 | 20 | Alb & AlbSO | 400 | NA | Oral Tablet (No Further Info) | All Males | NA (Adults) | NA | None | None |
| Chhonker [7] | 2018 | 1 | 7 | Alb & AlbSO | 400 | NA | Oral Tablet (No Further Info) | Mixture | NA (Adults) | None | IVM | Mixture (None;  Lymphatic filariasis) |
| Corti  [8] | 2009 | 3 | 24 | Alb & AlbSO | 400 | 5.48 | Oral Tablet (Brand=Zentel) | All Males | 31 (Adults) | None | Various (None; Ritonavir) | None |
| Cotting  [9] | 1990 | 3 | 3 | AlbSO Only | 200 | Various | Oral Tablet (No Further Info) | All Females | Various (Adults) | Fatty Meal | Various (Amoxicillin & Gentamicin; Metronidazole and Ceftriaxone) | Echinococcosis |
| Delatour [10] | 1991 | 1 | 4 | AlbSO Only | 725 | 10 | Oral (No Further Info) | All Males | Various (Adults) | None | None | None |
| Edi  [11] | 2019 | 2 | 56 | Alb & AlbSO | 400 | NA | Oral Tablet (Brand=Zentel) | Mixture | Various (Adults) | NA | IVM & DEC | Various (None;  Lymphatic filariasis) |
| Hoaksey [12] | 1991 | 2 | 32 | AlbSO Only | Various | Various | Oral Tablet (Brand=Smith Kline Beecham) | All Males | 37 (Adults) | Fatty Meal | None | Onchocerciasis |
| Hofmann [13] | 2022 | 11 | 253 | AlbSO Only | Various | Various | Oral Tablet (Brand=Zentel) | Mixture | Various (Chil-dren & Adults) | Fatty Meal | None | STH (Trichuriasis & Hookworm infection) |
| John  [14] | 2020 | 2 | 25 | Alb & AlbSO | 400 | Various | Oral Tablet (No Further Info) | Mixture | Various (Adults) | Unclear | Various (DEC & Ivermectin or DEC, Ivermectin & Azithromycin) | None |
| Jung  [15] | 1992 | 8 | 8 | AlbSO Only | Various | 15 | Oral Tablet (Brand=Zentel) | Mixture | Various (Adults) | Fasted | None | Neurocysticer-cosis |
| Jung  [16] | 1997 | 8 | 8 | AlbSO Only | Various | 15 | Oral Suspension (Brand=Zentel) | Mixture | Various (Chil-dren) | Fatty Meal | None | Neurocysticer-cosis |
| Kitzman [17] | 2002 | 1 | 1 | Alb & AlbSO | 400 | NA | Oral (No Further Info) | NA | NA | NA | None | None |
| Lange  [18] | 1988 | 2 | 12 | AlbSO Only | 400 | 5.65 | Oral Tablet (No Further Info) | Mixture | 43.5 (Adults) | Various | None | None |
| Monteiro [19] | 2010 | 2 | 18 | AlbSO Only | 400 | 6.23 | Oral Tablet (Brand=Zentel) | Mixture | 26 (Adults) | None | Various (None; PZQ) | None |
| Marriner [20] | 1986 | 10 | 10 | AlbSO Only | 400 | 5.93 | Oral Tablet (Brand=Zentel) | NA | 27.5 (Adults) | Various | None | None |
| Mingjie [21] | 2002 | 1 | 7 | AlbSO Only | 830 | 12.5 | Oral Tablet (No Further Info) | All Males | 29.3 (Adults) | NA | None | Echinococcosis |
| Mirfazaelian [22] | 2002 | 3 | 30 | AlbSO Only | Various | Various | Oral Tablet (No Further Info) | Mixture | 32.5 (Adults) | None | None | None |
| Mirfazaelian [23] | 2003 | 2 | 12 | AlbSO Only | 800 | 11.81 | Oral Tablet (No Further Info) | Mixture | 30 (Adults) | Fasted | None | None |
| Na-Bangchang [24] | 2006 | 2 | 46 | AlbSO Only | 400 | 7.53 | Oral Tablet (Brand=Zentel) | Mixture | 21 (Adults) | None | Various (IVM; IVM & PZQ) | None |
| Nagy  [25] | 2002 | 4 | 24 | AlbSO Only | 690 | 10 | Oral Capsule (Brand=Zentel) | All Males | 20 (Adults) | Various | Various (None; Cimetidine) | None |
| Ochoa  [26] | 2021 | 2 | 24 | Alb & AlbSO | 400 | 5.41 | Oral (No Further Info) | Mixture | 24.25 (Adults) | Various | None | None |
| Okelo  [27] | 1993 | 5 | 5 | AlbSO Only | 250 | 9.33 | Oral Tablet (Brand=Zentel) | All Males | 9.5 (Chil-dren) | NA | None | Echinococcosis |
| Pengsaa [28] | 2004 | 2 | 20 | Alb & AlbSO | 400 | Various | Oral Tablet (Brand=Zentel) | Mixture | Various (Children) | Fatty Meal | Various (None; PZQ) | Giardiasis |
| Rathod  [29] | 2016 | 1 | 51 | Alb & AlbSO | 400 | NA | Oral Tablet (Brand=Generic India) | NA | NA (Adults) | None | None | None |
| Rigter  [30] | 2004 | 1 | 1 | AlbSO Only | 400 | 7.14 | Oral Tablet (Brand=Zentel) | NA | 29.5 (Adults) | None | None | None |
| Sarin  [31] | 2004 | 1 | 10 | AlbSO Only | 600 | 10.03 | Oral (No Further Info) | NA | 32.5 (Adults) | Fatty Meal | None | None |
| Schipper [32] | 2000 | 9 | 30 | AlbSO Only | Various | Various | Oral Capsule (Brand=SmithKline Beecham) | All Males | 20 (Adults) | None | None | None |
| Schulz  [33] | 2019 | 1 | 10 | Alb & AlbSO | 400 | NA | Oral (No Further Info) | NA | 16.5 (Chil-dren) | None | Oxantel pamoate | STH (Hookworm infection) |
| Sergio- Mares [34] | 2005 | 2 | 32 | AlbSO Only | 800 | 12.72 | Oral Tablet (Brand=Eskazole) | Mixture | 24.7 (Adults) | Various | None | None |
| Shenoy  [35] | 2002 | 2 | 28 | Alb & AlbSO | 400 | Various | Oral Tablet (Brand=Smith Kline Beecham) | Mixture | 31.5 (Adults) | None | Various (None; DEC) | None |
| Thomsen [36] | 2016 | 2 | 24 | AlbSO Only | 400 | Various | Oral (No Further Info) | Mixture | Various (Adults) | None | Various (None; IVM & DEC) | Lymphatic filariasis |

Alb: Albendazole; AlbSO: Albendazole sulphoxide; IVM: ivermectin; DEC: diethylcarbamazine; PZQ: praziquantel; NA: not available.

**Supplementary Text B: Model Construction, Fitting and Inference**

***Mathematical Model of Albendazole and Albendazole Sulfoxide Pharmacokinetics***

A mathematical model describing the evolution of albendazole and albendazole sulfoxide concentrations in the plasma following receipt of a single dose, based on series of linked ordinary differential equations (ODEs) was developed. The model included a number of pharmacokinetic dynamics features known to be relevant to albendazole, including its limited bioavailability (which is thought to be due to its poor solubility along the gastrointestinal tract) [37] as well as first-pass metabolism of albendazole to albendazole sulfoxide known to occur via the liver [38]. In brief, following administration of an oral dose of albendazole, we model the amount of drug in the gut, and its subsequent absorption into the body. We model the newly absorbed albendazole as passing directly through a liver compartment that converts some proportion of passaged albendazole into the metabolite albendazole sulfoxide via first-pass metabolism. Subsequent circulation and exchange of peripheral and hepatic blood leads to further conversion of albendazole into albendazole sulfoxide. Additionally, we model both albendazole and albendazole sulfoxide as being metabolised by enzymatic processes, leading to gradual removal over time. The model is specified mathematically as follows,

| $\frac{d{Alb}_{G}}{dt}= -k_{Abs}{Alb}_{G}$ | Eqn. [S1] |
| --- | --- |
| $\frac{d[{Alb}_{L}]}{dt}= k_{Abs}{Alb}_{G}- \sigma\left[ {Alb}_{L} \right]-Q\left[ {Alb}_{L} \right]+Q[{Alb}_{P}]$ | Eqn. [S2] |
| $\frac{d[{Alb}_{P}]}{dt}= Q\left[ {Alb}_{L} \right]-Q\left[ {Alb}_{P} \right]-k_{Alb}[{Alb}_{P}]$ | Eqn. [S3] |
| $\frac{d[{AlbSO}_{P}]}{dt}= \sigma\left[ {Alb}_{L} \right]-k_{AlbSO}[{AlbSO}_{P}]$ | Eqn. [S4] |

where ${Alb}_{G}$, refers to the amount of albendazole in the gut; $[{Alb}_{L}]$ refers to the concentration of albendazole in the liver, $[{Alb}_{P}]$ the concentration of albendazole in the systemic circulation blood plasma, and $[{AlbSO}_{P}]$ the concentration of the metabolite albendazole sulfoxide in the systemic circulation blood plasma. $k_{Abs}$ is the rate of absorption of albendazole from the gut into the bloodstream (and implicitly includes a conversion translating the absolute amount of albendazole absorbed from the gut to the corresponding concentration in the liver compartment); $sigma$ denotes the rate at which albendazole is converted to albendazole sulfoxide by the liver; $k_{Alb}$ and $k_{AlbSO}$ are the rates at which albendazole and albendazole sulfoxide, respectively, are processed and cleared in the body; $Q$ represents the rate of exchange between the liver and the systemic blood plasma, and for the purposes of the results presented here is set to 15 (reflecting the approximate extent of exchange anticipated per hour between the two compartments [39]). The dose of albendazole used is further multiplied by another parameter, $systemic availability$ such that the initial amount of albendazole in the gut compartment (i.e. ${Alb}_{G}$) is given by the dose amount multiplied by $systemic availability$, which corresponds to the proportion of the albendazole dose received that is available for absorption and conversion to albendazole sulfoxide.

***Model Fitting and Inferential Framework***

The above pharmacokinetic mathematical model was fitted within a Bayesian framework. Specifically, the model was fitted to each dataset individually, using an adaptive Metropolis-Hastings Markov Chain Monte Carlo (MH-MCMC) sampling algorithm developed previously [40]. Weakly informative priors were set over $k_{Abs}$ and $sigma,$ (i.e. the two parameters where further inference and association with collated metadata in the form of multiple linear regression was not carried out). For the other parameters, $systemic availability$, $k_{Alb}$, and $k_{AlbSO}$, uninformative priors were used. Prior distributions for the estimated parameters were defined as follows,

$$k_{Abs} \sim Normal(Mean= 5, Variance=7)$$

$$systemic availability\sim Normal(Mean= 0,Variance= 1)$$

$$sigma \sim Normal(Mean= 15, Variance= 7)$$

$$k_{Alb} \sim Normal(Mean= 0, Variance= 1)$$

$$k_{AlbSO} \sim Normal(Mean= 0, Variance= 1)$$

truncated at 0 so that only positive parameter values were accepted. For both albendazole and albendazole sulfoxide plasma concentrations, a Poisson likelihood (reflecting the assumption that the drugs are well-mixed within each of our modelled compartments) was used, such that the model likelihood could be constructed as follows,

$$\left[ Alb \right]_{i}\sim Poisson\left( \left[ \tilde{Alb} \right]_{i} \right)$$

$$\left[ AlbSO \right]_{i}\sim Poisson\left( \left[ \tilde{AlbSO} \right]_{i} \right)$$

where $\left[ Alb \right]_{i}$ and $\left[ AlbSO \right]_{i}$ represent the empirically observed plasma concentrations of albendazole and albendazole sulfoxide respectively at time-point $i$. $\left[ \tilde{Alb} \right]_{i}$ and $\left[ \tilde{AlbSO} \right]_{i}$ represent the modelled plasma concentrations of albendazole and albendazole sulfoxide, respectively, at timepoint$i$. For each of the 92 time-series, a total of 50,000 iterations of the MCMC sampling algorithm were run for purposes of model fitting and parameter inference. Half of each chain’s iterations were discarded as burn-in/the adaptive phase of the sampling, leaving a total of 25,000 iterations available for inference.

***Pharmacokinetic Parameter Estimation and Multiple Linear Regression Modelling***

We next sought to associate the estimates of pharmacokinetic parameters obtained from fitting the above model, to collected metadata associated with each time-series (describing aspects of the patient population and treatment regimen received), to assess the influence of these factors on variation in albendazole and albendazole sulfoxide’s pharmacokinetics. These pharmacokinetic parameters were $k_{AlbSO}$ (related to the half-life of albendazole sulfoxide), the systemic availability of albendazole sulfoxide, $C_{Max}$ (the peak concentration of albendazole sulfoxide in the plasma) and $AUC$ (reflecting the total exposure to albendazole sulfoxide after administration of the dose, calculated over a time-period of 50 hours).

For each time-series, we calculated the median value of $systemic availability$ and $k_{AlbSO}$directly from the MCMC chains generated during model fitting. However, we were unable to calculate $C_{Max}$ and $AUC$ for albendazole sulfoxide directly from the fitted model output as studies differed substantially in the size of the dose administered (which would directly affect estimates of these two quantities). Therefore, we used the median estimates of each model parameter from the model fitting process described above, and for each time-series, simulated a hypothetical pharmacokinetic curve assuming a standardised dose of 400mg. From this hypothetical curve, standardised to have the same dose as all other time-series, we then calculated $C_{Max}$ and $AUC$; we subsequently refer to these quantities as $C_{Max400}$ and ${AUC}_{400}.$

Using a multiple linear regression-based approach, we then associated each of these pharmacokinetic parameters with the suite of collated individual/group metadata described in further detail in **Supplementary Table A**. When examining the impact of different specific infectious diseases, we replaced the infection status variable with binary indicators for STH (soil-transmitted helminthiases, caused by *Trichuris trichiura* or hookworm)/giardiasis (caused by *Giardia intestinalis*), onchocerciasis (caused by infection with *Onchocerca volvulus*), echinococcosis (caused by infection with *Echinococcus granulosus* or *E. multilocularis*) and neurocysticercosis (caused by infection with *Taenia solium* (where 1 indicates that individuals or group of individuals has that infection and 0 indicates an absence of the particular infection).

**Sensitivity Analysis**

As a sensitivity analysis, we repeated the multivariate linear regression analysis described above but this time by controlling for the dose of albendazole received per kilogram of body weight (available only for a subset of the time-series due to a lack of complete information about participants’ weight), rather than the raw amount (in mg, not standardised by body weight) given to an individual (**Supplementary Table B**).


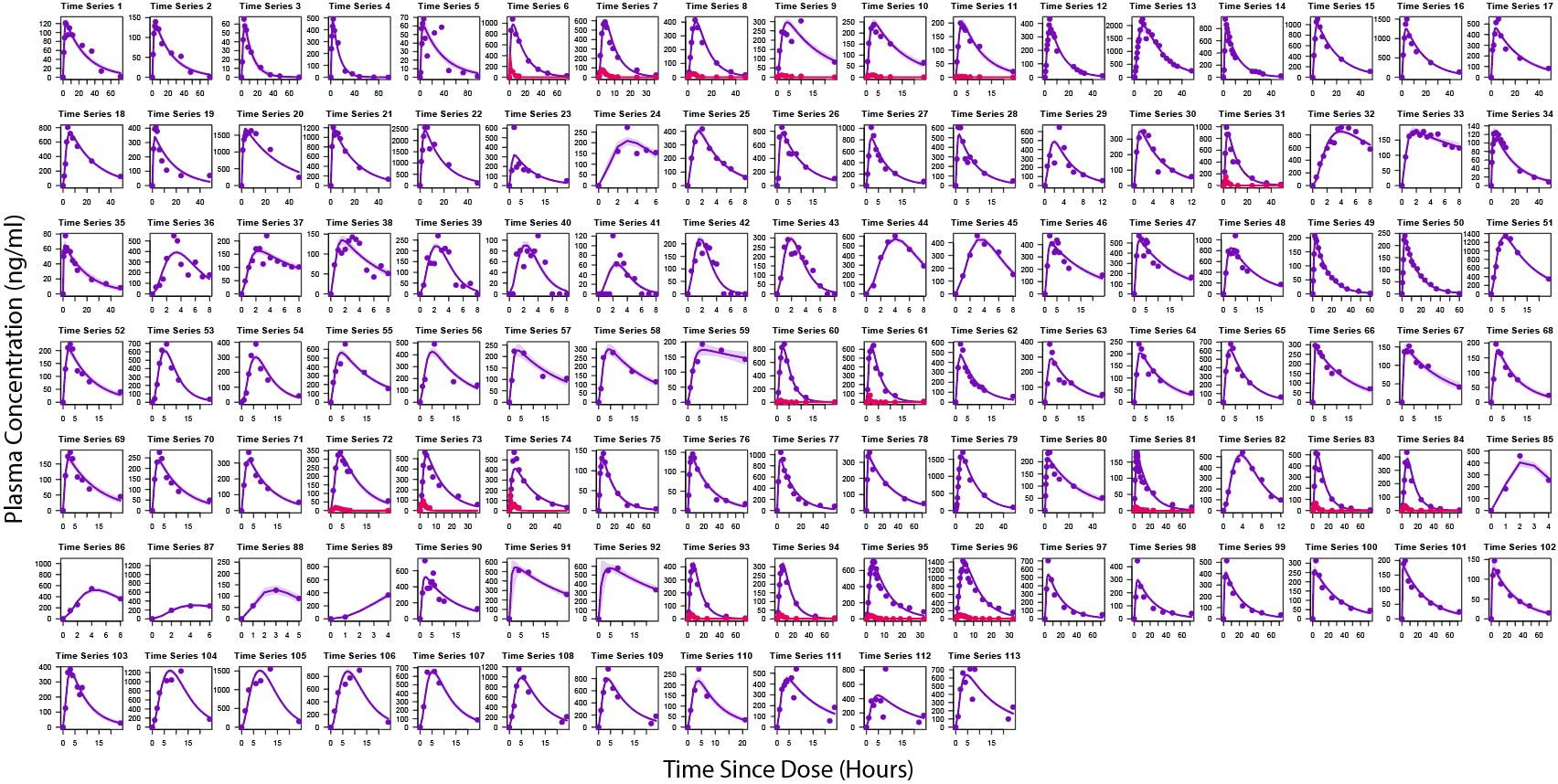


**Supplementary Figure A: Results of model fitting and calibration to data collated through the systematic review.** The systematic review identified a total of 113 time-series containing information on the plasma concentration of albendazole and/or albendazole sulfoxide following treatment with a single oral dose. The pharmacokinetic model of Eqns. [S1–S4] was fitted to these data individually using a Bayesian MCMC-based framework. This fitting was carried out in order to estimate the various pharmacokinetic parameters governing the model. For the results presented above, points represent empirical data and the lines represent model output, with the results for albendazole in pink and those for albendazole sulfoxide in purple. Pale shaded areas represent the 95% Bayesian Credible Interval.

**Supplementary Table B: Multiple linear regression results relating pharmacokinetic properties to study characteristics when controlling for dosage per kilogram of body weight instead of raw dosage amount in milligrams.** Inferred pharmacokinetic parameters, specifically systemic availability, albendazole sulfoxide clearance rate (reciprocal of the half-life), *C_Max_* and *AUC* were regressed onto various characteristics of the study populations controlling for sex, feeding status, age, dose per kilogram of body weight, presence of other infections (including breakdown by whether or not that infection is onchocerciasis, echinococcosis or neurocysticercosis) and co-administration of other drugs. Entries in green indicate statistical significance.

|  | **Bioavailability** | **AlbSO Clearance Rate** | ***AUC* (For Standardised 400mg Dose)** | ***C_Max_* (For Standardised 400mg Dose)** |
| --- | --- | --- | --- | --- |
| **Sex**  **(Male = Ref)** | p=0.15 | p=0.87 | p=0.46 | p=0.17 |
| **Fatty Meal** | p=0.02 | p=0.93 | p=0.01 | p=0.002 |
| **Age Group**  **(Adults = Ref)** | p=0.03 | p=0.01 | p=0.71 | p=0.01 |
| **Dose (Per kg body weight)** | p=0.93 | p=0.34 | p=0.36 | p=0.09 |
| **Parasitic Infection**  **(Ref = None)** | p=0.43 | p=0.15 | p=0.94 | p=0.40 |
| **🡺 Onchocerciasis** | p=0.65 | p=0.42 | p=0.86 | p=0.38 |
| **🡺 Echinococcosis** | p=0.09 | p=0.66 | p=0.002 | p=0.01 |
| **🡺 Neurocysticercosis** | p=0.06 | p=0.002 | p=0.75 | p=0.46 |
| **🡺 Soil-Transmitted Helminth Infection/Giardiasis/Lymphatic Filariasis** | p=0.15 | p=0.65 | p=0.04 | p=0.02 |
| **Co-Administered Drugs**  **(Ref = None)** | p=0.54 | p=0.89 | p=0.74 | p=0.44 |

**Supplementary References**

1. Awadzi K, Edwards G, Duke BOL, Opoku NO, Attah SK, Addy ET, et al. The co-administration of ivermectin and albendazole--safety, pharmacokinetics and efficacy against *Onchocerca volvulus*. Ann Trop Med Parasitol. 2003;97: 165–178.

2. Awadzi K, Edwards G, Opoku NO, Ardrey AE, Favager S, Addy ET, et al. The safety, tolerability and pharmacokinetics of levamisole alone, levamisole plus ivermectin, and levamisole plus albendazole, and their efficacy against *Onchocerca volvulus*. Ann Trop Med Parasitol. 2004;98: 595–614.

3. Awadzi K, Hero M, Opoku NO, Büttner DW, Coventry PA, Prime MA, et al. The chemotherapy of onchocerciasis XVII. A clinical evaluation of albendazole in patients with onchocerciasis; effects of food and pretreatment with ivermectin on drug response and pharmacokinetics. Trop Med Parasitol. 1994;45: 203–208.

4. Ceballos L, Krolewiecki A, Juárez M, Moreno L, Schaer F, Alvarez LI, et al. Assessment of serum pharmacokinetics and urinary excretion of albendazole and its metabolites in human volunteers. PLoS Negl Trop Dis. 2018;12: e0005945.

5. Ceballos L, Nieves E, Juárez M, Aveldaño R, Travacio M, Martos J, et al. Assessment of diet-related changes on albendazole absorption, systemic exposure, and pattern of urinary excretion in treated human volunteers. Antimicrob Agents Chemother. 2021;65: e0043221.

6. Chen X, Zhao L, Xu H, Zhong D. Simultaneous determination of albendazole and its major active metabolite in human plasma using a sensitive and specific liquid chromatographic-tandem mass spectrometric method. J Pharm Biomed Anal. 2004;35: 829–836.

7. Chhonker YS, Edi C, Murry DJ. LC–MS/MS method for simultaneous determination of diethylcarbamazine, albendazole and albendazole metabolites in human plasma: application to a clinical pharmacokinetic study. J Pharm Biomed Anal. 2018;151: 84–90.

8. Corti N, Heck A, Rentsch K, Zingg W, Jetter A, Stieger B, et al. Effect of ritonavir on the pharmacokinetics of the benzimidazoles albendazole and mebendazole: an interaction study in healthy volunteers. Eur J Clin Pharmacol. 2009;65: 999–1006.

9. Cotting J, Zeugin T, Steiger U, Reichen J. Albendazole kinetics in patients with echinococcosis: delayed absorption and impaired elimination in cholestasis. Eur J Clin Pharmacol. 1990;38: 605–608.

10. Delatour P, Benoit E, Besse S, Boukraa A. Comparative enantioselectivity in the sulphoxidation of albendazole in man, dogs and rats. Xenobiotica. 1991;21: 217–221.

11. Edi C, Bjerum CM, Ouattara AF, Chhonker YS, Penali LK, Méité A, et al. Pharmacokinetics, safety, and efficacy of a single co-administered dose of diethylcarbamazine, albendazole and ivermectin in adults with and without *Wuchereria bancrofti* infection in Côte d’Ivoire. PLoS Negl Trop Dis. 2019;13: e0007325.

12. Hoaksey PE, Awadzi K, Ward SA, Coventry PA, Orme ML, Edwards G. Rapid and sensitive method for the determination of albendazole and albendazole sulphoxide in biological fluids. J Chromatogr. 1991;566: 244–249.

13. Hofmann D, Brussee JM, Schulz JD, Coulibaly JT, Pfister M, Keiser J. Pharmacokinetic modelling and simulation to optimize albendazole dosing in hookworm- or *Trichuris trichiura*-infected infants to adults. J Antimicrob Chemother. 2022;77: 1082–1093.

14. John LN, Bjerum C, Martinez PM, Likia R, Silus L, Wali C, et al. Pharmacokinetic and safety study of co-administration of albendazole, diethylcarbamazine, ivermectin and azithromycin for the integrated treatment of Neglected Tropical Diseases. Clin Infect Dis. 2020. doi:10.1093/cid/ciaa1202

15. Jung H, Hurtado M, Sanchez M, Medina MT, Sotelo J. Clinical pharmacokinetics of albendazole in patients with brain cysticercosis. J Clin Pharmacol. 1992;32: 28–31.

16. Jung H, Sánchez M, González-Astiazarán A, Martínez JM, Suástegui R, González-Esquivel DF. Clinical pharmacokinetics of albendazole in children with neurocysticercosis. Am J Ther. 1997;4: 23–26.

17. Kitzman D, Cheng K-J, Fleckenstein L. HPLC assay for albendazole and metabolites in human plasma for clinical pharmacokinetic studies. J Pharm Biomed Anal. 2002;30: 801–813.

18. Lange H, Eggers R, Bircher J. Increased systemic availability of albendazole when taken with a fatty meal. Eur J Clin Pharmacol. 1988;34: 315–317.

19. Lima RM, Ferreira MAD, de Jesus Ponte Carvalho TM, Dumêt Fernandes BJ, Takayanagui OM, Garcia HH, et al. Albendazole-praziquantel interaction in healthy volunteers: kinetic disposition, metabolism and enantioselectivity. Br J Clin Pharmacol. 2011;71: 528–535.

20. Marriner SE, Morris DL, Dickson B, Bogan JA. Pharmacokinetics of albendazole in man. Eur J Clin Pharmacol. 1986;30: 705–708.

21. Mingjie W, Shuhua X, Junjie C, Bin L, Cheng F, Weixia S, et al. Albendazole-soybean oil emulsion for the treatment of human cystic echinococcosis: evaluation of bioavailability and bioequivalence. Acta Trop. 2002;83: 177–181.

22. Mirfazaelian A, Rouini MR, Dadashzadeh S. Dose dependent pharmacokinetics of albendazole in human. Biopharm Drug Dispos. 2002;23: 379–383.

23. Mirfazaelian A, Rouini MR, Dadashzadeh S. Time dependent pharmacokinetics of albendazole in human. Biopharm Drug Dispos. 2003;24: 199–204.

24. Na-Bangchang K, Kietinun S, Pawa KK, Hanpitakpong W, Na-Bangchang C, Lazdins J. Assessments of pharmacokinetic drug interactions and tolerability of albendazole, praziquantel and ivermectin combinations. Trans R Soc Trop Med Hyg. 2006;100: 335–345.

25. Nagy J, Schipper HG, Koopmans RP, Butter JJ, Van Boxtel CJ, Kager PA. Effect of grapefruit juice or cimetidine coadministration on albendazole bioavailability. Am J Trop Med Hyg. 2002;66: 260–263.

26. Ochoa D, Saiz-Rodríguez M, González-Rojano E, Román M, Sánchez-Rojas S, Wojnicz A, et al. High-fat breakfast increases bioavailability of albendazole compared to low-fat breakfast: single-dose study in healthy subjects. Front Pharmacol. 2021;12: 664465.

27. Okelo GB, Hagos B, Ng’ang’a JN, Ogeto JO. Pharmacokinetics of albendazole in children with hydatid disease. East Afr Med J. 1993;70: 643–645.

28. Pengsaa K, Na-Bangchang K, Limkittikul K, Kabkaew K, Lapphra K, Sirivichayakul C, et al. Pharmacokinetic investigation of albendazole and praziquantel in Thai children infected with *Giardia intestinalis*. Ann Trop Med Parasitol. 2004;98: 349–357.

29. Rathod DM, Patel KR, Mistri HN, Jangid AG, Shrivastav PS, Sanyal M. Liquid chromatography--tandem mass spectrometry method for simultaneous determination of albendazole and albendazole sulfoxide in human plasma for bioequivalence studies. J Pharm Anal. 2016;6: 226–234.

30. Rigter IM, Schipper HG, Koopmans RP, van Kan HJM, Frijlink HW, Kager PA, et al. Relative bioavailability of three newly developed albendazole formulations: a randomized crossover study with healthy volunteers. Antimicrob Agents Chemother. 2004;48: 1051–1054.

31. Sarin R, Dash AP, Dua VK. Albendazole sulphoxide concentrations in plasma of endemic normals from a lymphatic filariasis endemic region using liquid chromatography. J Chromatogr B Analyt Technol Biomed Life Sci. 2004;799: 233–238.

32. Schipper HG, Nagy J, Van Boxtel CJ, Koopmans RP, Kager PA, Butter JJ. Effect of dose increase or cimetidine co-administration on albendazole bioavailability. Am J Trop Med Hyg. 2000;63: 270–273.

33. Schulz JD, Neodo A, Coulibaly JT, Keiser J. Pharmacokinetics of albendazole, albendazole sulfoxide, and albendazole sulfone determined from plasma, blood, dried-blood spots, and Mitra samples of hookworm-infected adolescents. Antimicrob Agents Chemother. 2019;63. doi:10.1128/AAC.02489-18

34. Mares SS, Jung CH, López AT, González-Esquivel DF. Influence of a Mexican diet on the bioavailability of albendazole. Basic Clin Pharmacol Toxicol. 2005;97: 122–124.

35. Shenoy RK, Suma TK, John A, Arun SR, Kumaraswami V, Fleckenstein LL, et al. The pharmacokinetics, safety and tolerability of the co-administration of diethylcarbamazine and albendazole. Ann Trop Med Parasitol. 2002;96: 603–614.

36. Thomsen EK, Sanuku N, Baea M, Satofan S, Maki E, Lombore B, et al. Efficacy, safety, and pharmacokinetics of coadministered diethylcarbamazine, albendazole, and ivermectin for treatment of Bancroftian filariasis. Clin Infect Dis. 2016;62: 334–341.

37. Jung H, Medina L, García L, Fuentes I, Moreno-Esparza R. Absorption studies of albendazole and some physicochemical properties of the drug and its metabolite albendazole sulphoxide. J Pharm Pharmacol. 1998;50: 43–48.

38. Lawrenz A, Eglit S, Kroker R. The metabolism of albendazole in the isolated perfused intestine of rats. Dtsch Tierarztl Wochenschr. 1992;99: 416–418.

39. Eipel C, Abshagen K, Vollmar B. Regulation of hepatic blood flow: the hepatic arterial buffer response revisited. World J Gastroenterol. 2010;16: 6046–6057.

40. Johnstone RH, Chang ETY, Bardenet R, de Boer TP, Gavaghan DJ, Pathmanathan P, et al. Uncertainty and variability in models of the cardiac action potential: Can we build trustworthy models? J Mol Cell Cardiol. 2016;96: 49–62.
