# Supplementary material for: Factors associated with variation in single-dose albendazole pharmacokinetics: A systematic review and modelling analysis
Source: PLoS Negl Trop Dis. 2022 Oct 28;16(10):e0010497. doi: 10.1371/journal.pntd.0010497 (PMC9662735; doi:10.1371/journal.pntd.0010497)
Supplement: Attachment — Submitted filename: Revised Whittaker Alb PK Replies to Referees.docx [file pntd.0010497.s002.docx]

We thank all of the reviewers for their insightful and helpful comments, which we feel have materially improved the manuscript and made the presented results more robust. Enclosed below is a point-by-point explanation of how we have addressed and incorporated these comments into the revised version of the manuscript. Thanks again! **__________________________________________________________________________**

**Methods**

-Are the objectives of the study clearly articulated with a clear testable hypothesis stated?

-Is the study design appropriate to address the stated objectives?

-Is the population clearly described and appropriate for the hypothesis being tested?

-Is the sample size sufficient to ensure adequate power to address the hypothesis being tested?

-Were correct statistical analysis used to support conclusions?

-Are there concerns about ethical or regulatory requirements being met?

**Reviewer #1:** Did you do a PROSPERO registration?
The authors should update their literature search as three years old! I had a quick search on Pubmed and there are a number of relevant studies, which should be included (e.g. the study by Hofmann has >250 participants, which is higher than all of the studies reported in your work. It is even mentioned as limitation in the discussion section….

We thank the reviewer for raising this important point and entirely agree with their assessment that the literature review we previously carried required updating. We have now done this, extending the searches from 4^th^ July 2019 to 22^nd^ July 2022. A total of 1165 new abstracts were identified, of which 40 were taken to full-text review and 4 of which were subsequently included. These were:

*John LN, Bjerum C, Martinez PM, Likia R, Silus L, Wali C, et al. "Pharmacokinetic and safety study of co-administration of albendazole, diethylcarbamazine, ivermectin and azithromycin for the integrated treatment of Neglected Tropical Diseases." Clin Infect Dis. 2020;* *ciaa1202. doi: 10.1093/cid/ciaa1202. Online ahead of print.*

*Ochoa D, Saiz-Rodríguez M, González-Rojano E, Román M, Sánchez-Rojas S, Wojnicz A, et al. "High-fat breakfast increases bioavailability of albendazole compared to low-fat breakfast: Single-dose study in healthy subjects." Front Pharmacol. 2021;12: 664465.*

*Hofmann D, Brussee JM, Schulz JD, Coulibaly JT, Pfister M, Keiser J. "Pharmacokinetic modelling and simulation to optimize albendazole dosing in hookworm- or Trichuris trichiura-infected infants to adults." J Antimicrob Chemother. 2022;77: 1082-1093.*

*Ceballos L, Nieves E, Juárez M, Aveldaño R, Travacio M, Martos J, et al. "Assessment of diet-related changes on albendazole absorption, systemic exposure, and pattern of urinary excretion in treated human volunteers." Antimicrob Agents Chemother. 2021;65: e00432-21.*

which together contained an additional 21 albendazole sulfoxide plasma time-series following receipt of a single oral dose, bringing the total number of collated time-series used in our analyses from n=92 to n=113 (an increase of over 20%). Relevant data and metadata for each of these pharmacokinetic time-series have been extracted, and all analyses in the paper are now based on this significantly expanded set of collated time-series. We did not do a PROSPERO registration ahead of carrying out the systematic review.

**Reviewer #2:** The main objectives are clear, and the inclusion criteria used to perform the systematic review are defined in the test. Authors developed a pharmacokinetic model within a Bayesian framework adaptive Metropolis-Hastings based Markov Montecarlo which is commonly used for population models.

**Reviewer #3:** Please see comments below

**__________________________________________________________________________**

**Results**

-Does the analysis presented match the analysis plan?

-Are the results clearly and completely presented?

-Are the figures (Tables, Images) of sufficient quality for clarity?

**Reviewer #1:** I would add the references of the included studies in the main paper not only in the appendix Table 1: add soil-transmitted helminths

We thank the reviewer for their comments here. We have gone through and added references for all of the included studies into the main paper (rather than just in the Supplementary Material) and have additionally included details of whether or not individuals had a soil-transmitted helminth infection. In the previous search carried out, only a single pharmacokinetic study carried out in individuals with a soil-transmitted helminth infection (hookworm) was identified; however, one of the references newly identified in the updated search also explored albendazole sulfoxide pharmacokinetics in patients infected either with *Trichuris* or hookworm. We have, therefore, added this study into Supplementary Table 1 and updated it so that all studies where individuals had infections with soil-transmitted helminths are clearly indicated, as well as included STH/*Giardia* as a covariate in the analyses examining the role of infection with these pathogens on pharmacokinetics.

**Reviewer #2:** Figures and tables are clear and meet the quality requirements

**Reviewer #3:** See my specific comments below

**__________________________________________________________________________**

**Conclusions**

-Are the conclusions supported by the data presented?

-Are the limitations of analysis clearly described?

-Do the authors discuss how these data can be helpful to advance our understanding of the topic under study?

-Is public health relevance addressed?

**Reviewer #1:** Line 343: how does the model compare with a previously published pop PK model of albendazole?

We thank the reviewer for this comment, and have added text in the relevant part of the Discussion to better situate this work in the context of previously published pharmacokinetic models of albendazole and albendazole sulfoxide, with a particular focus on highlighting the similarity of this model to previously published ODE-based models.

*“Indeed, whilst the overall modelling framework used here is similar to previously published models of albendazole sulfoxide pharmacokinetics (e.g. the use of explicit gut, peripheral and central compartments to capture relevant properties of albendazole’s absorption and pharmacokinetics [71]), there are a number of additions to this framework that would likely provide new insight into albendazole sulfoxide’s pharmacokinetics.”*

where reference 71 is:

*Turner JD, Sharma R, Al Jayoussi G, Tyrer HE, Gamble J, Hayward L, et al. "Albendazole and antibiotics synergize to deliver short-course anti-Wolbachia curative treatments in preclinical models of filariasis." Proc Natl Acad Sci USA. 2017;114: E9712-E9721.*

Line 366: yes, this was described earlier. Please place your results in context with previous work.

The reviewer is completely correct that receipt of a fatty meal and its influence on albendazole/ albendazole sulfoxide pharmacokinetics and, in particular, on systemic availability has been previously documented. To that end, we have added the following text into the relevant part of the Discussion, along with the following references highlighting the previous description and establishment of this phenomenon:

*“For example, whilst receipt of a fatty meal was associated with increases to systemic availability, AUC_400_ and C_Max400_ (in agreement with previous work exploring its effect [27,30])…”*

Where references are:

*27. Lange H, Eggers R, Bircher J. Increased systemic availability of albendazole when taken with a fatty meal. Eur J Clin Pharmacol. 1988;34: 315–317.*

*30. Ceballos L, Nieves E, Juárez M, Aveldaño R, Travacio M, Martos J, et al. Assessment of diet-related changes on albendazole absorption, systemic exposure, and pattern of urinary excretion in treated human volunteers. Antimicrob Agents Chemother. 2021;65: e0043221.*

Line 369: I do not find this very interesting as this has been reported earlier (T. trichiura infection was for example found to be correlated with lower clearance of both albendazole metabolites)

We thank the reviewer for their comment. In (Hofmann et al., J Antimicrob Chemother. 2022), we found a similar phrase to that used by the reviewer above, which we assume they are referring to: *“Interestingly, differences were found between individuals infected with the two different STH species. A* ***T. trichiura infection was found to be correlated with lower clearance of both metabolites****, which we hypothesize may be due to possible differences in uptake of the drug in the intestine, rather than different elimination rates.”* However, in that study, the authors compared pharmacokinetics of albendazole and albendazole sulfoxide in subjects infected with hookworms compared to *T. trichiura*, not between subjects infected with *T. trichiura* and healthy subjects.

Line 401: there is no paucity of data, just update your literature search….

We thank the reviewer for their comments (here and elsewhere, which we feel have improved the manuscript significantly), and we agree that updating the literature search has significantly increased the amount of available data to analyse. We have removed the text previously present at Line 401 in response to this point.

**Reviewer #2:** Conclusions should state the most important outcomes of the manuscript. The interindividual variability has been recognized for a long time.

We thank the reviewer for this comment. We have adjusted the text to reflect this comment, and the other comment from Reviewer #2 below (*“Authors highlight the existence of a significant interindividual variation, however these results are very well known and have been previously reported in most of the articles, therefore conclusions should be oriented to the advance that authors found in order to understand the variability.”*). Note that we dedicate the entire second and third paragraphs of the Discussion section to detailing the conclusions of this work with regards to the factors that contribute to this variability.

**Reviewer #3:** See comments below

**__________________________________________________________________________**

**Editorial and Data Presentation Modifications?**

Use this section for editorial suggestions as well as relatively minor modifications of existing data that would enhance clarity. If the only modifications needed are minor and/or editorial, you may wish to recommend “Minor Revision” or “Accept”.

Reviewer #1: Abstract

This variation is thought to have important consequences for treatment success, please specify the disease that this was demonstrated

We thank the reviewer for this suggestion and agree with their comment that the evidence surrounding the role of inter-individual variation in albendazole sulfoxide pharmacokinetics and its impact on treatment efficacy is nascent and often quite limited. To our knowledge, there is no direct work highlighting the consequences of inter-individual pharmacokinetic variation on treatment success, though one line of evidence supporting this assertion comes from work suggesting that higher doses of albendazole are often associated with better treatment success rates – this includes results from patients with neurocysticercosis (<https://academic.oup.com/cid/article/69/11/1996/5306011?login=false#supplementary-data>) and also from individuals infected with hookworm, both in studies of escalating single doses (<https://pubmed.ncbi.nlm.nih.gov/32668456/>), and in trials where single doses were compared to three doses delivered daily (<https://journals.plos.org/plosone/article?id=10.1371/journal.pone.0025003>). A corollary of higher doses being associated with treatment efficacy is that high levels of inter-individual variation in albendazole sulfoxide pharmacokinetics (resulting in large variation in the systemic availability of albendazole sulfoxide and hence systemic exposure) would contribute to variation in treatment efficacy. We completely agree with the reviewer’s assessment, however, that this has not been definitively shown, and have therefore modified the abstract to couch this statement in more tentative terms, to accurately reflect the more limited direct evidence supporting this claim:

*“This variation might have implications for the efficacy of albendazole treatment, but current understanding of the factors associated with this variation remains incomplete.”*

We have additionally adapted and modified the text in the Introduction that discusses variation in treatment success – the details of these amendments are described in response to the comment below.

Line 37: STH infections were also shown to alter the PK of albendazole, hence this should be added here

We apologise to the reviewer if this was unclear, but our initial results did not show evidence of STH infection altering pharmacokinetic dynamics (indeed our original search only identified a single reference where pharmacokinetics of albendazole sulfoxide had been assessed in individuals infected with an STH, hookworm specifically (Schulz et al., Antimicrob Agents Chemother. 2019). Updating the systematic review, we identified an additional study reporting pharmacokinetic results for individuals infected with STH (Hofmann et al., J Antimicrob Chemother. 2022). Data paucity precluded including STH infection as a standalone covariate (instead being combined with LF and giardiasis) and did not reveal a significant association with any pharmacokinetic parameters in the main text analysis and so we have not added text to this effect in the Abstract. There was suggestion of a significant effect in the sensitivity analyses carried out and displayed in the SI, and we have added text in the Results section describing this.

Introduction

Line 92: and other confounding factors could be infection intensity or diagnostic method etc.. As for hookworm intestinal concentrations might be more relevant this is not an ideal example to mention that PK variations of albendazole have an influence on treatment success. This is pure speculation and has not been demonstrated

We thank the reviewer for their comment. Building on the response given two comments above (about existence of data we consider supportive but lack of definitive evidence), we have amended this section to couch it in more tentative terms and have modified it to focus instead on the results linking higher albendazole doses with improved treatment efficacy, and given this phenomenon, how significant inter-individual pharmacokinetic variability could shape treatment success rates between individuals. We agree with their suggestion about removing the more speculative elements of the text and the rewritten section now reads:

*“Previous work has highlighted a relationship between higher albendazole sulfoxide blood plasma (henceforth referred to as plasma) levels and increased antiparasitic efficacy in patients with neurocysticercosis [17]. Similarly, higher dosages of albendazole have been associated with higher efficacy against hookworm [18], as have utilisation of triple-dose regimens over single-dose ones [19]. In this context, substantial inter-individual pharmacokinetic variability might contribute to the failure of cure observed in some treated patients. Indeed, across both individual treatment contexts in healthcare facilities [13,20,21] and field studies [22], highly variable impact of the drug on clearing treated infections has been observed. However, some of these observations might also be driven by other factors, such as variation between settings in the prevalence of different STH species which are thought to show variable responses to albendazole treatment [23].“*

Where references are:

*13. Jung-Cook H. Pharmacokinetic variability of anthelmintics: implications for the treatment of neurocysticercosis. Expert Rev Clin Pharmacol. 2012;5: 21–30.*

*17. Arroyo G, Bustos JA, Lescano AG, Gonzales I, Saavedra H, Rodriguez S, et al. Albendazole sulfoxide plasma levels and efficacy of antiparasitic treatment in patients with parenchymal neurocysticercosis. Clin Infect Dis. 2019;69: 1996–2002.*

*18. Patel C, Coulibaly JT, Hofmann D, N’Gbesso Y, Hattendorf J, Keiser J. Efficacy and safety of albendazole in hookworm-infected preschool-aged children, school-aged children, and adults in Côte d’Ivoire: A phase 2 randomized, controlled dose-finding trial. Clin Infect Dis. 2021;73: e494–e502.*

*19. Steinmann P, Utzinger J, Du Z-W, Jiang J-Y, Chen J-X, Hattendorf J, et al. Efficacy of single-dose and triple-dose albendazole and mebendazole against soil-transmitted helminths and Taenia spp.: a randomized controlled trial. PLoS One. 2011;6: e25003.*

*20. Del Brutto OH. Albendazole therapy for subarachnoid cysticerci: clinical and neuroimaging analysis of 17 patients. J Neurol Neurosurg Psychiatry. 1997;62: 659–661.*

*21. García HH, Evans CAW, Nash TE, Takayanagui OM, White AC Jr, Botero D, et al. Current consensus guidelines for treatment of neurocysticercosis. Clin Microbiol Rev. 2002;15: 747–756.*

*22. Mrus J, Baeten B, Engelen M, Silber SA. Efficacy of single-dose 500 mg mebendazole in soil-transmitted helminth infections: a review. J Helminthol. 2018;92: 269–278.*

*23. Basáñez MG, French MD, Walker M, Churcher TS. Paradigm lost: how parasite control may alter pattern and process in human helminthiases. Trends Parasitol. 2012;28: 161–171.*

Reviewer #2: (No Response)

Reviewer #3: (No Response)

**__________________________________________________________________________**

**Summary and General Comments**

Use this section to provide overall comments, discuss strengths/weaknesses of the study, novelty, significance, general execution and scholarship. You may also include additional comments for the author, including concerns about dual publication, research ethics, or publication ethics. If requesting major revision, please articulate the new experiments that are needed.

**Reviewer #1:** Whittaker and colleagues have done a systematic review on albendazole and PK. This is a well written paper and an interesting analysis. I agree with the authors that limited data is available on pharmacokinetics on NTD drugs and this is a topic to be addressed. However, the study has an important limitation since the literature search is not up to date and therefore the authors missed recent studies, which will have an influence on their results.

We thank the reviewer for this important comment. As described in the response at the beginning of these comments, we completely agree with their assessment, and have updated the review so that the time-period searched now goes up to 22^nd^ July 2022. The results of this time-period expansion have been collated and integrated into all the analyses, now updated and present in the revised manuscript presented here.

**Reviewer #2:** The main contribution of the manuscript is the effect of the parasitic infection on Albendazole Pharmacokinetics. Authors highlight the existence of a significant interindividual variation, however these results are very well known and have been previously reported in most of the articles, therefore conclusions should be oriented to the advance that authors found in order to understand the variability.

- Authors state that the results correspond to albendazole or albendazole sulfoxide concentrations in blood. Most of the analytical assays use plasma for the quantification of the drug and its metabolites. Please explain

We apologise for the confusion and lack of clarity in the text. The reviewer is completely correct that our usage of blood was unspecific, incorrect and that the intended description was of plasma. We reviewed all the extracted references and can confirm blood plasma was used in all instances. We have therefore amended the text and used “plasma” instead of blood.

- In figure 2, differential equations assume that albendazole is absorbed in the gut, however it has been shown that the drug undergoes extensive intestinal bioconversion. If the equation were included in the model, would you expect a change in the results obtained? Please explain

We thank the reviewer for this comment. Given the extensive and rapid first-pass metabolism of albendazole to albendazole sulfoxide in the liver that also occurs (and which is included in the model structure used in our analyses), explicit consideration of intestinal bioconversion (which would also contribute to the phenomenon of rapid conversion and limited albendazole plasma levels observed) would be unlikely to change the results presented here. However, we agree entirely with the reviewer that this is an important consideration, and to that end have added the following text to the Discussion highlighting this as a limitation of the model presented here:

*“…there are a number of additions to this framework that would likely provide new insight into albendazole sulfoxide’s pharmacokinetics. These include the intestinal bioconversion of albendazole to albendazole sulfoxide known to occur [72], as well as infrequent but reported pharmacokinetic phenomena associated with albendazole treatment, such as biphasic pharmacokinetic profiles (thought possibly to be a product of inter-individual variation in frequency of gastric emptying affecting release of ingested albendazole into the gut, as well as other related characteristics….”*

Where Reference 72 is:

*Villaverde C, Alvarez AI, Redondo P, Voces J, Del Estal JL, Prieto JG. "Small intestinal sulphoxidation of albendazole." Xenobiotica. 1995;25: 433-441.*

- Line 243. Paragraph: Cmax value is 354 mg/ml higher or almost twice as high in individuals taking a fatty meal. is not clear. Albendazole sulfoxide levels are reported in micrograms/mL and not in mg/mL. Sentence should be clarified

We apologise for this error on our part. The units should be in ng/ml, as with the rest of results presented in the manuscript. We have amended and corrected this error.

- Different authors have shown that one of the main causes of interindividual variability is due to differences in gastric pH or intestinal metabolism. No mention about the gastric pH was included in the manuscript.

We thank the reviewer for this comment and apologise for this important omission. We have gone back through the manuscript and added text detailing the relevance of this phenomenon, as well as cited studies that highlight 1) the general pH-dependent absorption of albendazole/albendazole sulfoxide; 2) evidence for pH-dependent absorption in animal models and 3) the same phenomenon in humans. We have amended text in the Introduction as follows:

*“This variation has been consistently observed across a wide range of studies (see [13] for a review and its implications for treatment). It is typically attributed to the drug’s limited solubility in the gastrointestinal tract (thought to be driven, in part, by inter-individual variability in gastric pH and intestinal metabolism, which can significantly impact the drug’s absorption and bioavailability [14–16]) as well as extensive first-pass metabolism by the liver (responsible for rapid conversion of albendazole to albendazole sulfoxide).”*

Where the new references 14-16 are:

*14. Schipper HG, Koopmans RP, Nagy J, Butter JJ, Kager PA, Van Boxtel CJ. Effect of dose increase or cimetidine co-administration on albendazole bioavailability. Am J Trop Med Hyg. 2000;63: 270–273.*

*15. Jung H, Medina L, García L, Fuentes I, Moreno-Esparza R. Absorption studies of albendazole and some physicochemical properties of the drug and its metabolite albendazole sulphoxide. J Pharm Pharmacol. 1998;50: 43–48.*

*16. Kohri N, Yamayoshi Y, Iseki K, Sato N, Todo S, Miyazaki K. Effect of Gastric pH on the Bioavailability of Albendazole in Rabbits. Pharm Pharmacol Commun. 1998;4: 267–270.*

and in the Discussion:

*“Relatedly, whilst we were able to explore the association of some factors with albendazole sulfoxide pharmacokinetic variability, information on other factors known to influence the pharmacokinetics of albendazole and albendazole sulfoxide (such as inter-individual variation in gastric pH which can significantly influence the drug’s absorption given the pH dependence of its solubility [14–16]) was not present in the collated references, and so we were unable to quantify its impact on albendazole and albendazole sulfoxide pharmacokinetics.“*

**Reviewer #3:** I’ve now completed the revision of the manuscript (PNTD-D-22-00636) entitled “Factors associated with variation in single-dose albendazole pharmacokinetics: A systematic review and modelling analysis”, which has been submitted for publication in PNTD. The manuscript describes the results obtained from a systematic review addressed to identify literature references containing temporally disaggregated data on the blood/plasma concentration profiles of albendazole and its main active metabolite albendazole suphoxide following a single oral dose. These data were then integrated into a mathematical modelling to infer key pharmacokinetic parameters and relate them to population features (age, sex, diet etc) which could affect drug pharmacokinetics and anthelmintic efficacy. This is a well written and presented article which may result of interest to the PNTD readership. It´s well known the key role of albendazole in MDA based STH control programs. Additionally, it is well established (since long time ago) that a number of host and drug formulation related factors may drastically affect albendazole/metabolites pharmacokinetic behavior and its resultant anthelmintic efficacy in different animal species and in man. Thus, the data obtained from the current systematic review is useful to highlight the relevance of the extensive inter-individual variation on albendazole pharmacokinetics which may explain often therapeutic failures against common STH species. Overall, this may be a useful contribution to the specific field since ratifies in a summarized format (using systematic review and modeling tools) well established principles in anthelmintic pharmacology. However, the article requires revision in many different aspects before it can be recommended for publication in PNTD. The following issues should be specifically addressed:

1.- The literature search was based on the Web of Science and PubMed databases until July 2019 using the keywords “albendazole” AND (treatment* OR dose* OR pharma* OR “half-life” OR “half life”) in order to identify references related to the main topics. Although a total of 5690 records were identified through this search process, only 32 studies containing temporally disaggregated data on drug/metabolite blood concentrations following treatment with a single dose were used to run the proposed model. Since research on albendazole and its performance on STH control programs is a very dynamic scientific field, I´d strongly suggest the authors to extend the systematic literature search until the current year. It sounds “deficient” and “incomplete” (outdated) to publish a literature search-based work whose cut-off line for incorporating data is 3 years old.

We thank the reviewer for raising this important point and entirely agree with their assessment that the literature review we previously carried required updating. We have now done this, extending the searches from 4^th^ July 2019 to 22^nd^ July 2022. A total of 1165 new abstracts were identified, of which 40 were taken to full-text review and 4 of which were subsequently included. These were:

*John LN, Bjerum C, Martinez PM, Likia R, Silus L, Wali C, et al. "Pharmacokinetic and safety study of co-administration of albendazole, diethylcarbamazine, ivermectin and azithromycin for the integrated treatment of Neglected Tropical Diseases." Clin Infect Dis. 2020;* *ciaa1202. doi: 10.1093/cid/ciaa1202. Online ahead of print.*

*Ochoa D, Saiz-Rodríguez M, González-Rojano E, Román M, Sánchez-Rojas S, Wojnicz A, et al. "High-fat breakfast increases bioavailability of albendazole compared to low-fat breakfast: Single-dose study in healthy subjects." Front Pharmacol. 2021;12: 664465.*

*Hofmann D, Brussee JM, Schulz JD, Coulibaly JT, Pfister M, Keiser J. "Pharmacokinetic modelling and simulation to optimize albendazole dosing in hookworm- or Trichuris trichiura-infected infants to adults." J Antimicrob Chemother. 2022;77: 1082-1093.*

*Ceballos L, Nieves E, Juárez M, Aveldaño R, Travacio M, Martos J, et al. "Assessment of diet-related changes on albendazole absorption, systemic exposure, and pattern of urinary excretion in treated human volunteers." Antimicrob Agents Chemother. 2021;65: e00432-21.*

which together contained an additional 21 albendazole aulfoxide plasma time-series following receipt of a single oral dose, bringing the total number of collated time-series used in our analyses from n=92 to n=113 (an increase of over 20%). Relevant data and metadata for each of these pharmacokinetic time-series have been extracted, and all analyses in the paper are now based on this significantly expanded set of collated time-series.

2. There is a main issue to be corrected throughout the whole manuscript and fully address in a revised version of the article. Albendazole suffers a very efficient first-pass metabolism in the liver (and intestinal mucosa). Its sulphoxide and sulphone metabolites are the main albendazole related molecules recovered in the bloodstream. Only trace amounts of albendazole parent drug are occasionally recovered in the bloodstream in most of the animal species and man treated with a single oral dose. Although using modern chromatographic technology (HPLC MS/MS with very low detection sensitivity) it may be possible to detect “trace” albendazole concentrations in blood, any sound pharmacokinetic analysis can be performed for the parent drug. Traditionally, the estimations on albendazole systemic availability have been indirectly based on the availability of its main sulphoxide (active) and sulphone (inactive) metabolites. Thus, it’s unsound to refer to parent albendazole blood concentration profiles and even more inaccurate, to establish any conclusion on variations on its pharmacokinetic behavior. Could the authors check in how many of the 32 studies being incorporated was albendazole parent drug recovered in the bloodstream?

We thank the reviewer for their comment and apologise for a lack of clarity throughout the manuscript. Whilst our model simulates both albendazole and albendazole pharmacokinetic dynamics (and explicitly incorporates the extensive first-pass metabolism that occurs in the liver), all of the downstream analyses around pharmacokinetic behaviour and factors associated with its variation relate to albendazole sulfoxide, not albendazole. Results surrounding bioavailability (now renamed to “systemic availability” per the reviewer’s comment below), *C_Max_*_­_, *AUC* and half-life relate to albendazole sulfoxide, not albendazole. Indeed, the pharmacokinetics of albendazole sulfoxide is the focus of this work (for the reasons the reviewer has noted above).

Across the collated pharmacokinetic studies, 94/113 total studies contained information on albendazole sulfoxide concentrations in plasma over time only, and 19/113 studies contained information on both albendazole and albendazole sulfoxide concentrations in plasma over time. Where information on both were available, we fitted the model to both; where only albendazole sulfoxide data were presented, the model was fitted to albendazole sulfoxide data only. Whilst model fitting used the maximum amount of data available and thus sometimes included albendazole data where these were presented, downstream analyses of inferred model parameters were restricted to considering those for albendazole sulfoxide only. For example, we examine and analyse variation in the inferred half-life and systemic availability of albendazole sulfoxide, but not for albendazole. The conclusions in the paper therefore relate to variation in albendazole sulfoxide’s pharmacokinetic behaviour, not albendazole’s. We completely agree, however, that this was unclear and have amended all instances in the text where we informally use “albendazole” or “the drug” when in fact the quantity we are referring to is “albendazole sulfoxide” (which is the subject of model inference and all presented analyses in the paper).

On this reviewer´s opinion, the outcome of the current manuscript should be only focused on the identification of sources of variation to the pharmacokinetics of the active metabolite (albendazole sulphoxide) which can be recovered in the bloodstream and tissues of parasite location, and it´s the best “indicator” of albendazole absorption and systemic availability. The data shown in Figure 3 (Albendazole sulfoxide pharmacokinetic variability, stratified by patient and dosage features) is the most solid outcome of the performed work and should be a main focus of the revised version of the manuscript. To clarify and reinforce this main outcome of the reported systematic review and modelling (effect of fatty diet), the authors should revise recently published data on the impact of diet on albendazole sulphoxide systemic exposure in healthy volunteers treated with oral albendazole. The following references emerging from our work on those topics, as well as others available in the literature, should be illustrative of all the above described arguments.

-Assessment of serum pharmacokinetics and urinary excretion of albendazole and its metabolites in human volunteers. Ceballos L, Krolewiecki A, Juárez M, Moreno L, Schaer F, Alvarez LI, Cimino R, Walson J, Lanusse CE. PLoS Negl Trop Dis. 2018 Jan 18;12(1):e0005945. doi: 10.1371/journal.pntd.0005945. eCollection 2018 Jan.

-Assessment of Diet-Related Changes on Albendazole Absorption, Systemic Exposure, and Pattern of Urinary Excretion in Treated Human Volunteers. Ceballos L, Nieves E, Juárez M, Aveldaño R, Travacio M, Martos J, Cimino R, Walson JL, Krolewiecki A, Lanusse C, Alvarez L. Antimicrob Agents Chemother. 2021 Aug 17;65(9):e0043221. doi: 10.1128/AAC.00432-21. Epub 2021 Aug 17.

We thank the reviewer for their comment and agree with their assessment that the work should focus on the pharmacokinetics of the active metabolite albendazole sulfoxide. This is indeed the focus of our work, which we hope is now clearer thanks to the changes to the text we have made in light of the reviewers’ suggestions. We have additionally, per this comment, included the above references to text in the Introduction and Discussion, highlighting the relevance and importance of fatty meal delivery to albendazole sulfoxide pharmacokinetics. We have, therefore, updated the text (whereas the previous references were from 2005 or earlier) with these more contemporary reports. We also note that both of these studies were identified in the literature review we carried out (Ceballos et al. 2018 in our original search and Ceballos et al. 2021 in our updated search associated with this manuscript revision). Both references feature in the analyses presented in the revision of our work.

3. In the light of above raised comments, it´s likely the authors may need to fully revise some of the ODEs (equations) governing the model applied here.

We thank the reviewer for this comment, but in light of the clarification provided above – specifically, that all the model pharmacokinetic parameters analysed in the paper relate to albendazole sulfoxide (and not albendazole), we do not feel that any revision of the ODEs is required.

4. There are some “terms” throughout the manuscript that should be revised to avoid confusion. The most of them are related to well established pharmacological terminology that sometimes is incorrectly used. The term “bioavailability” (absolute bioavailability) of a given drug can only be used when the plasma concentration profiles obtained by an extravascular route has been compared with those obtained after the intravenous administration. Systemic availability and/or systemic exposure are correct terms to refer to the amount of the administered drug reaching the bloodstream. Thus, is inaccurate to express the results and interpretation of your modeling as ….. " bioavailability of albendazole" and "AUC of albendazole sulfoxide". This is wrong, confusing and should be fully clarified. Otherwise, the interpretation and outcome of the systematic review and modeling is misleading.
Additionally, pharmacokinetics and pharmacodynamics are well established definitions and extensively used terms in Pharmacology. The authors should be careful on how they use some terminology in the manuscript. Expressions such as “A number of factors are deemed to underlie this variation in pharmacokinetic dynamics (line 93), “we fitted a mathematical model of albendazole and albendazole sulfoxide’s dynamics in the blood following receipt of the dose that…(line 105)”, “….. our work highlights the extensive inter individual pharmacokinetic variation in albendazole’s dynamics (line 345). Please avoid use the term “dymanics” when you refer to pharmacokinetic issues/variables. This type of corrections should be applied both in the text, figure legends etc, to avoid confusion and misunderstanding.

We again apologise for the lack of clarity here, and appreciate the reviewer’s input on how best to improve terminology. We have now gone through the manuscript and, per the reviewer’s comments, have made corrections and amendments where relevant to sharpen the language used and make it more precise in terms of the exact phenomena being described. Specifically, we have renamed “bioavailability” as “systemic availability of albendazole sulfoxide” in all instances. We have also removed all references to “dynamics” and instead refer to “pharmacokinetics” in all instances.

5. It has also been well established that the type drug formulation (preparation) may drastically affect the pattern of albendazole (and related benzimidazole compounds) dissolution at the stomach acidic pH and the resultant GI absorption and systemic availability of the sulphoxide metabolite. This is a critical issue for the interpretation of the work reported here. Are the authors sure that in all the 32 studies included in the analysis, albendazole was administered under the same tablet formulation? Was the same commercially available oral formulation used in all the trials? What about dosing? The studies were all performed with fixed albendazole doses (400 mg) or in some of them there was a dose calculation expressed in mg/kg body weight? This type of information should be provided if available or expressed as serious limitations on the reported data.

We thank the reviewer for their comment and completely agree this is an important and relevant point. We have gone through each of the references and systematically collected information on the formulation used in each study (where available) and added this as a column in Supplementary Table 1. Across the collated studies, 28 provided information on the formulation used, with 25 being a standard oral table, 2 being with oral capsule and 1 with oral suspension) indicating that there wasn’t substantial variation in the formulation used between studies. We note that Supplementary Table 1 also contains information on the dosages used in each study, expressed both in mg and mg/kg body weight. Full information on dosing for each individual time-series is available in the supplementary data file; the openly accessible metadata are contained on the Github repository associated with this work: <https://github.com/cwhittaker1000/albendazole_pk/blob/main/Data/metadata_for_import.csv>.

6. Finally, the identification of sources of variation accounting for changes on albendazole dissolution, absorption and clinical performance can only be indirectly estimated by the systemic availability (exposure) and disposition kinetics of its main sulphoxide metabolite. The identification of host-related factors affecting the kinetic behavior of the main active albendazole metabolite, has been useful to establish strategies to improve albendazole anthelmintic performance avoiding common therapeutic failures both in Human and Veterinary Medicine. In many occasions, treatment failures are due to the incorrect use of the drug and not to genetic-based development of drug resistance as it´s very often attributed. For this reason, this reviewer strongly suggests the authors to correct the manuscript as recommended, considering this work is leaving a “useful take home message” on the need of further work to improve the use of albendazole (and other similar agents) in MDA campaigns for STH control.

We thank the reviewer for all of their incredibly helpful and insightful comments on this work, which have significantly improved it! All amendments and corrections proposed by the reviewer in all of their above comments have been addressed, and the work is much better for it – thank you!

**__________________________________________________________________________**
